# Sound localization and auditory selective attention in school-aged children with ADHD

**DOI:** 10.3389/fnins.2022.1051585

**Published:** 2022-12-15

**Authors:** Tong Fu, Bingkun Li, Weizhen Yin, Shitao Huang, Hongyu Liu, Yan Song, Xiaoli Li, Herui Shang, Yanling Zhou, Daomeng Cheng, Liping Cao, Cai-Ping Dang

**Affiliations:** ^1^The Affiliated Brain Hospital of Guangzhou Medical University, Guangzhou Medical University, Guangzhou, China; ^2^Department of Applied Psychology, Guangzhou Medical University, Guangzhou, China; ^3^Institute of Psychiatry and Psychology, The Affiliated Brain Hospital of Guangzhou Medical University, Guangzhou, China; ^4^State Key Laboratory of Cognitive Neuroscience and Learning and IDG/McGovern Institute for Brain Research, Beijing Normal University, Beijing, China; ^5^Center for Collaboration and Innovation in Brain and Learning Sciences, Beijing Normal University, Beijing, China

**Keywords:** ADHD, children, ERP, auditory selective attention, lateralization, N2ac

## Abstract

This study aimed to identify the neurophysiologic bases of auditory attention deficits in children with attention-deficit/hyperactivity disorder (ADHD), focusing on the electroencephalography component of auditory spatial selective attention [the N2 anterior contralateral component (N2ac)]. EEG data were collected from 7- to 11-year-old children with ADHD (*n* = 54) and age-, sex-, and IQ-matched typically developing (TD) children (*n* = 61), while they performed an auditory spatial selective task. For behavior, the children with ADHD showed a shorter reaction time (RT) but a higher RT coefficient of variability (RT_CV_) than TD children. For ERPs, the TD group showed a significant “adult-like” N2ac component; however, the N2ac component was absent in children with ADHD. More importantly, the smaller N2ac component could predict longer RT in both groups, as well as higher severity of inattentive symptoms in children with ADHD. Our results indicated that 7- to 11-year-old TD children have developed an “adult-like” ability to balance auditory target selection and distractor suppression; the absence of N2ac in children with ADHD provided novel evidence supporting their dysfunctional auditory spatial selective attention.

## Introduction

Our daily environment contains abundant auditory information that needs to be processed selectively. Imagine a situation where children concentrate on listening to the teacher in the class, accompanied by the abrupt whispers in the classroom or the sharp chirping of birds outside the classroom. For school-aged children, it is critical to select important information and ignore the distractors, and it is the main pre-condition for learning. Previous studies suggested that children achieved adult-level selective attention functions due to the interactions between the developing sensory cortices and frontoparietal control network in cluttered visual scenes (see review in Kim and Kastner, 2019). For EEG studies, children had smaller posterior contralateral N2 (N2pc) and larger compensatory distractor positivity (P_D_) than adults in visual spatial attention, suggesting insufficient attentional selection resources to targets but compensatory “adult-like” attentional suppression resources to resist irrelevant distractors (Sun et al., 2018). Meanwhile, previous studies reported smaller difference negativity (Nd) than adults (Berman and Friedman, 1995) in multiple auditory sequences. However, how children locate and balance task-relevant sounds and ignore irrelevant sounds remain unclear.

Attention-deficit/hyperactivity disorder (ADHD), characterized by core symptoms of age-inappropriate inattention, hyperactivity, and/or impulsivity (American Psychiatric Association, 2013; Peisch et al., 2021), is one of the most common neurodevelopmental disorders. According to previous studies, the estimated prevalence varied under different diagnostic criteria (Polanczyk et al., 2007, 2014), and the studies using only the DSM found the prevalence to be 7.2% (95%CI: 6.7–7.8%, Thomas et al., 2015); however, other studies with more criteria suggested the prevalence was 5.29% (95% CI: 5.01–5.56%, Polanczyk et al., 2007). Some researchers have reported that children with ADHD have normal selective attention (Mason et al., 2003; Huang-Pollock et al., 2005; McAvinue et al., 2015; Suarez et al., 2021); however, other researchers have found that children with ADHD have impaired visual selective attention (López et al., 2006; Ortega et al., 2013; Wang et al., 2016). Particularly, in auditory selective attention studies, some researchers regarded that children with ADHD had deficits in selective attention that reduced early perceptual processing in unilateral auditory stimuli (Gomes et al., 2012), which mainly resulted from problems in auditory selective filtering (Jonkman et al., 1997), while others argued that they only had dysfunctional attentional engagement instead of deficiency in auditory selective attention (Laffere et al., 2021). Therefore, it is still debated whether and how the impairment of auditory selective attention occurs in children with ADHD.

Previous studies have provided elementary evidence for the impairment mechanism of auditory selective attention in general. Auditory selective attention could alter the representation of sounds in the auditory cortex (Woldorff et al., 1993; Lee et al., 2014), and the lateral prefrontal cortex (LPFC) was involved in the control of the facilitatory mechanisms of auditory attention (Bidet-Caulet et al., 2015). For example, active frontotemporal networks were mainly observed on the contralateral hemisphere of the attended auditory compared with the ipsilateral hemisphere in dichotic listening tasks (Jäncke and Shah, 2002). Some MEG/fMRI studies in patients with ADHD found abnormal brain activity with auditory attention tasks (Heinrichs-Graham et al., 2014; Serrallach et al., 2016; Salmi et al., 2018, 2020). Furthermore, some EEG components have been investigated to provide an understanding of the impairment of auditory selective attention that occurs in children with ADHD. Mismatch negativity (MMN) has typically been observed in various EEG studies with common auditory oddball or auditory Go/NoGo tasks, reflecting the automatic detection of unpredictable audio. The ADHD studies on MMN were extensive but controversial, reporting both reduced MMN amplitude in children with ADHD (Rothenberger et al., 2000; Cheng et al., 2016; Yamamuro et al., 2016; Zhang et al., 2020) and comparable MMN amplitudes in ADHD studies of children (Huttunen et al., 2007; Gomes et al., 2013), adolescents (Rydkjær et al., 2017), and adults (le Sommer et al., 2021), compared to the healthy controls. Meanwhile, it remains controversial whether and how the extent of selective attention is involved in MMN (Haroush et al., 2010; Fishman, 2014), especially regarding patients with ADHD. In addition, the effect of Nd was still debated in individuals with ADHD, which reflected the attentive detection of the current stimulus feature in the channel of interest. Some studies reported no significantly impaired Nd effect in patients with ADHD (Rothenberger et al., 2000; Itagaki et al., 2011), while others found a smaller Nd effect in children with ADHD than in typically developing (TD) children (Satterfield et al., 1988; Jonkman et al., 1997; Gomes et al., 2012). These controversies might reflect the changes in MMN and Nd effects at different attentional levels of different tasks but cannot reflect the spatial shift of auditory attention. Therefore, it is worth exploring other ERP components that are more closely and directly related to auditory selective attention to investigate the deficiency of auditory selective attention in children with ADHD.

Recently, the EEG component of anterior contralateral N2 (N2ac), as a general electrophysiological marker of the lateralized focusing of auditory attention toward a detected target sound in an auditory scene, was observed at the anterior contralateral electrode sites 200–300 ms after the stimulus onset in the N2 latency (Gamble and Luck, 2011; Gamble and Woldorff, 2015b; Klatt et al., 2018). The N2ac component was analogous to the component of N2pc in visual attention studies, which counteracted the effects of physical stimulation asymmetry; furthermore, N2ac was the difference in lateralized task-related and task-unrelated auditory deviants. It also reflected the dynamics of spatial attention orienting in an auditory scene (Gamble and Luck, 2011). Further studies using variant paradigms in adults (Gamble and Woldorff, 2015a; Lewald and Getzmann, 2015; Lewald et al., 2016; Burra et al., 2019; Cai et al., 2020; Getzmann et al., 2020) suggested that the N2ac could be elicited independently by auditory targets, which reflected an initial, model-specific process of attentional lateralization (Klatt et al., 2018). In these studies, both target and non-target could induce lateralized components, and the contralateral-minus-ipsilateral difference waveform in the target was significantly higher than that of non-target; thus, the additional difference wave of target minus non-target leaving the N2ac component could better reflect the ability of lateralized task-related auditory deviants' detection (Gamble and Woldorff, 2015b). Furthermore, the large N2ac amplitude was accompanied by increased efficiency in selecting auditory spatial information in adults (Klatt et al., 2020). Studies of older adults observed comparable N2ac to younger adults, suggesting that older adults still had normal initial sound localization and attentional reorientation (Getzmann et al., 2020; Klatt et al., 2020). To date, no study focusing on the N2ac component in children has been reported; and the N2ac could provide a better understanding of auditory attention allocation in children with ADHD.

With an adapted three-stimulus oddball paradigm (Gamble and Woldorff, 2015b), this study aimed to investigate the following: (1) whether the N2ac component, as an index of auditory selective spatial attention, could be observed in children. If so, (2) were there significant differences in N2ac between TD children and children with ADHD? Furthermore, considering the previous EEG studies in visual selective attention (Cross-Villasana et al., 2015; Wang et al., 2016) and the MEG/fMRI studies in auditory attention (Heinrichs-Graham et al., 2014; Serrallach et al., 2016; Salmi et al., 2018, 2020), we hypothesized that there might be differences in the N2ac component associated with auditory spatial selective attention between the two groups. If so, we would examine (3) whether the dysfunctional N2ac component was related to core symptom severity in children with ADHD.

## Methods

### Participants

A total of 151 children (79 children with ADHD and 72 TD children) participated in the current study. Data from 36 participants (25 children with ADHD, 11 TD children) were excluded because of an accuracy of <50% or a high ratio of noise (and/or excessive eye movement and head movement) in the EEG signals. Thus, the remaining 115 participants (54 children with ADHD and 61 TD children) were included in the current study. There was no significant difference between the groups in terms of age, sex, or FSIQ (Table 1, *p*s > 0.144). Children with ADHD were recruited at the Affiliated Brain Hospital of Guangzhou Medical University. TD children were recruited from local schools. This study was approved by the Ethics Committee of the Affiliated Brain Hospital of Guangzhou Medical University. Informed consent was obtained from one parent of every child, and all research was carried out in accordance with the Declaration of Helsinki.

**Table 1:** Participant characteristics.

	**ADHD (*n* = 54)**	**TD (*n* = 61)**	**χ^2^/*t***	***df***	***p***
Sex (male: female)	47: 7	46: 15	2.503	1	0.114
Age (years)	9.99 ± 1.98	9.67 ± 1.48	0.988	97	0.326
FSIQ (standardized score)	108.98 ± 14.99	110.61 ± 12.50	−0.634	113	0.528
**Symptom (mean** **±SD)**					
SNAP-IV_inatt_	1.74 ± 0.55	0.70 ± 0.39	11.569	94	0.000
SNAP-IV_hyper_	1.28 ± 0.72	0.17 ± 0.19	10.980	60	0.000
SNAP-IV_full_	1.51 ± 0.57	0.44 ± 0.23	13.057	68	0.000

ADHD, children with attention-deficit/hyperactivity disorder; TD, TD children; FSIQ, full-scale intelligence quotient; SNAP-IV_*inatt*_, inattention subscale of SNAP-IV; SNAP-IV_*hyper*_, hyperactivity/impulsivity subscale of SNAP-IV; SNAP-IV_*full*_, full scale of SNAP-IV; SD, standard deviation.

All of the participants and their primary caretakers (usually the mothers) were interviewed using semi-structured diagnostic interviews (Kiddie-SADS-Lifetime Version, K-SADS-PL) (Kaufman et al., 1997) by a qualified psychiatrist to preliminarily confirm the tendency of ADHD symptoms. In addition, children with ADHD simultaneously met the full criteria from the *DSM-V* (Diagnostic and Statistical Manual of Mental Disorders, 5th edition) and *SNAP-IV* (Swanson, Nolan, and Pelham, Version IV Rating Scale), while TD children did not. Children with ADHD were requested to interrupt stimulant medicine 24 h before the experiment. All participants met the following inclusion criteria in this study: (*a*) normal hearing, (*b*) normal or corrected-to-normal vision, (*c*) no comorbidities such as schizophrenia, mood disorder, autism spectrum disorders, or epilepsy, (*d*) no history of head trauma with a loss of consciousness, (*e*) no history of organic diseases, neurological illness, or other severe diseases, (*f*) right-handedness, and (*g*) normal FSIQ (*Chinese WISC-IV full-scale IQ* > 80) (Wechsler, 2003).

### Materials and stimuli

**Figure 1:**
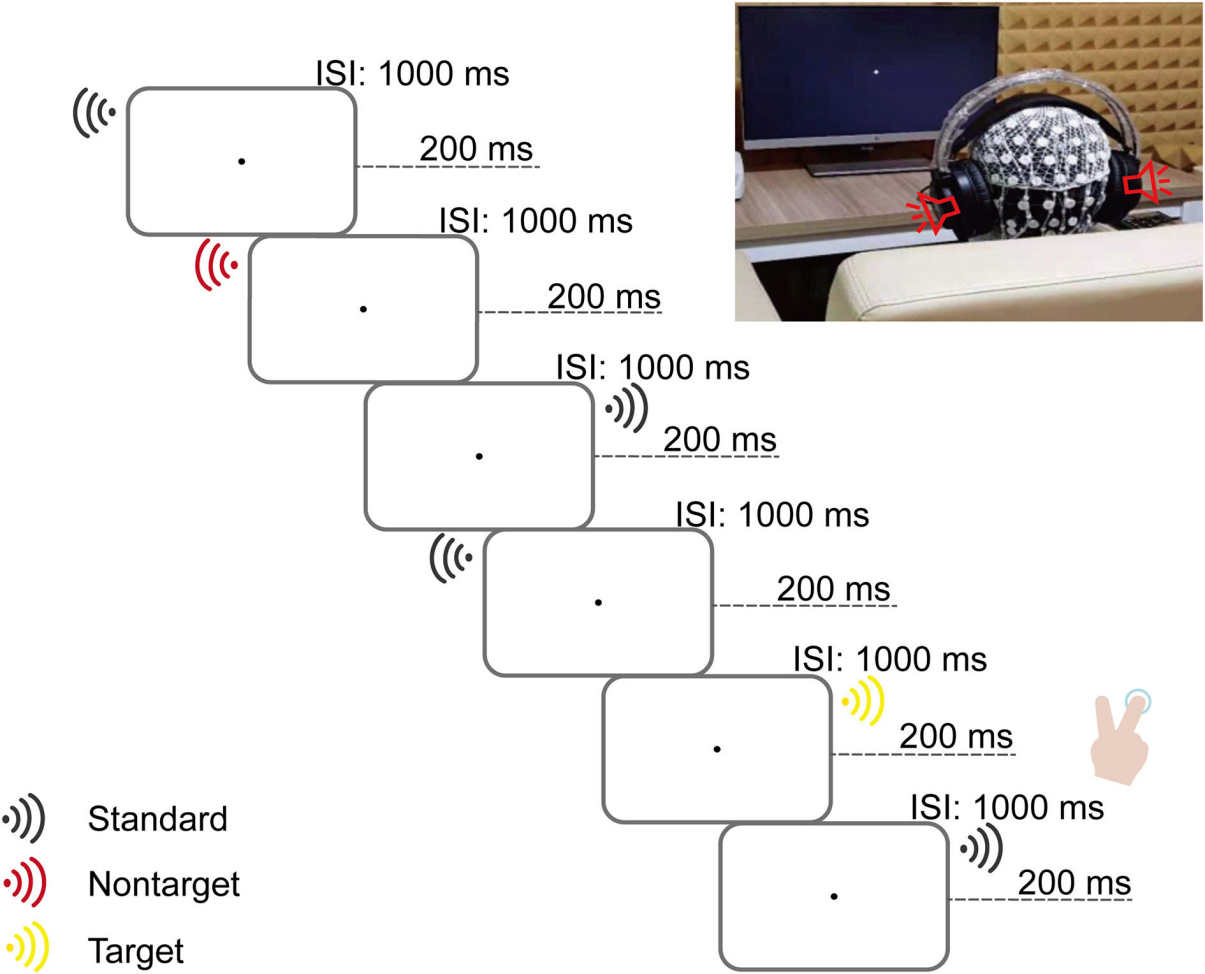
Illustration of a typical sequence of stimuli and a scene of a real experiment. In the **(Left)** panel, the flowchart shows a slice of the stimulus sequence. One stimulus was pseudorandomly played at a time in mono *via* a headphone (either the right or left side). Three types of stimuli were defined: target deviants (16.3% occurrence, 240 trials divided evenly on both sides) and unattended deviants (16.3% occurrence, 240 trials divided evenly on both sides) counterbalanced with the two tones (200 and 1,000 Hz) between subjects and non-target standards (67.4% occurrence, 992 trials divided evenly on both sides). Each trial with a duration of 1,200 ms consisted of one stimulus played for 200 ms, followed by an interstimulus interval (ISI) of 1,000 ms. In the **(Right)** panel, children were instructed to keep their eyes on the fixation point on a computer screen; meanwhile, they were instructed to determine the side of the target deviants (pressing the button “2” or “3” on the keyboard with the index or middle finger of the right hand if the target deviants played on the left or right side) as accurately and quickly as possible and ignore the other stimulus.

This auditory search task was adapted based on the three-stimulus oddball paradigm (Gamble and Woldorff, 2015b) and presented with E-Prime software (Version 2.0, Psychology Software Tools, Inc., USA). This is illustrated in Figure 1.

Three pure tone stimuli (200 Hz, 600 Hz, and 1,000 Hz with 200 ms duration) were generated with Praat software (Version 5.3.64, Paul Boersma and David Weenink, Phonetic Sciences Department, University of Amsterdam, the Netherlands) and were presented ~65 dB in headphones (HP GH10 Wired Gaming Headphones).

### Procedure and task

The task consisted of eight blocks within 1,472 trials in total. On each trial, only one stimulus lasted 200 ms with an interstimulus interval (ISI) of 1,000 ms, yielding a total trial duration of 1,200 ms, pseudorandomly played in mono *via* a headphone (either the right or left side). Thus, the trials were defined by three stimulus types: target deviants (16.3% occurrence, 240 trials divided evenly on both sides), non-target deviants (16.3% occurrence, 240 trials divided evenly on both sides), and non-target standards (67.4% occurrence, 992 trials divided evenly on both sides). Trials with target deviants and non-target deviants counterbalanced with the two tones (200 Hz and 1,000 Hz) between subjects. Trials with non-target standards used the other tone (600 Hz). Trials were pseudorandomly presented according to the following rules: (1) no more than four stimuli played successively on the same side; (2) no more than three identical stimuli played successively; (3) after every 1–3 non-target standard stimuli, there were randomly presented (non)target deviants; and (4) each block of stimulus sequence started and ended with non-target standards. Participants were provided with a break of ~2 min between blocks. After practice, the task lasted ~45 min.

Participants were instructed to sit comfortably ~70 cm away from the computer screen in a dimly lit, sound-attenuated, and electrically shielded cabin and to keep their eyes on the fixation point on the computer screen throughout the experiment. They were required to distinguish the side of the target deviants (by pressing the button “2” or “3” on the keyboard with the index or middle finger of the right hand (handedness) if the target deviants played on the left or right side) as accurately and quickly as possible.

### Data analysis

#### Behavioral data

Behaviorally, the reaction time (RT), RT coefficient of variability (RT_CV_), and ERROR were calculated for each participant. Among them, RT_CV_ (standard deviation of RT/mean RT) reflected the variability and deviation in response, which is known to be more sensitive than RT in the field of children with ADHD (Karalunas et al., 2014; Tye et al., 2016). ERROR was the total error number, which was more intuitive than accuracy due to no response most of the time as needed.

#### EEG recording and pre-processing

When the participants performed the auditory task, their EEG signals were synchronously collected with the Net Amps 400 amplifier (Electrical Geodesics, Inc., Eugene, OR) using the 64- or 128-channel system (Hydrocel Geodesic Sensor Net). The impedance of all electrodes was kept below 50 kΩ, and all electrodes were referenced physically to the Cz electrode at a sampling rate of 1,000 Hz during recording.

**Figure 2:**
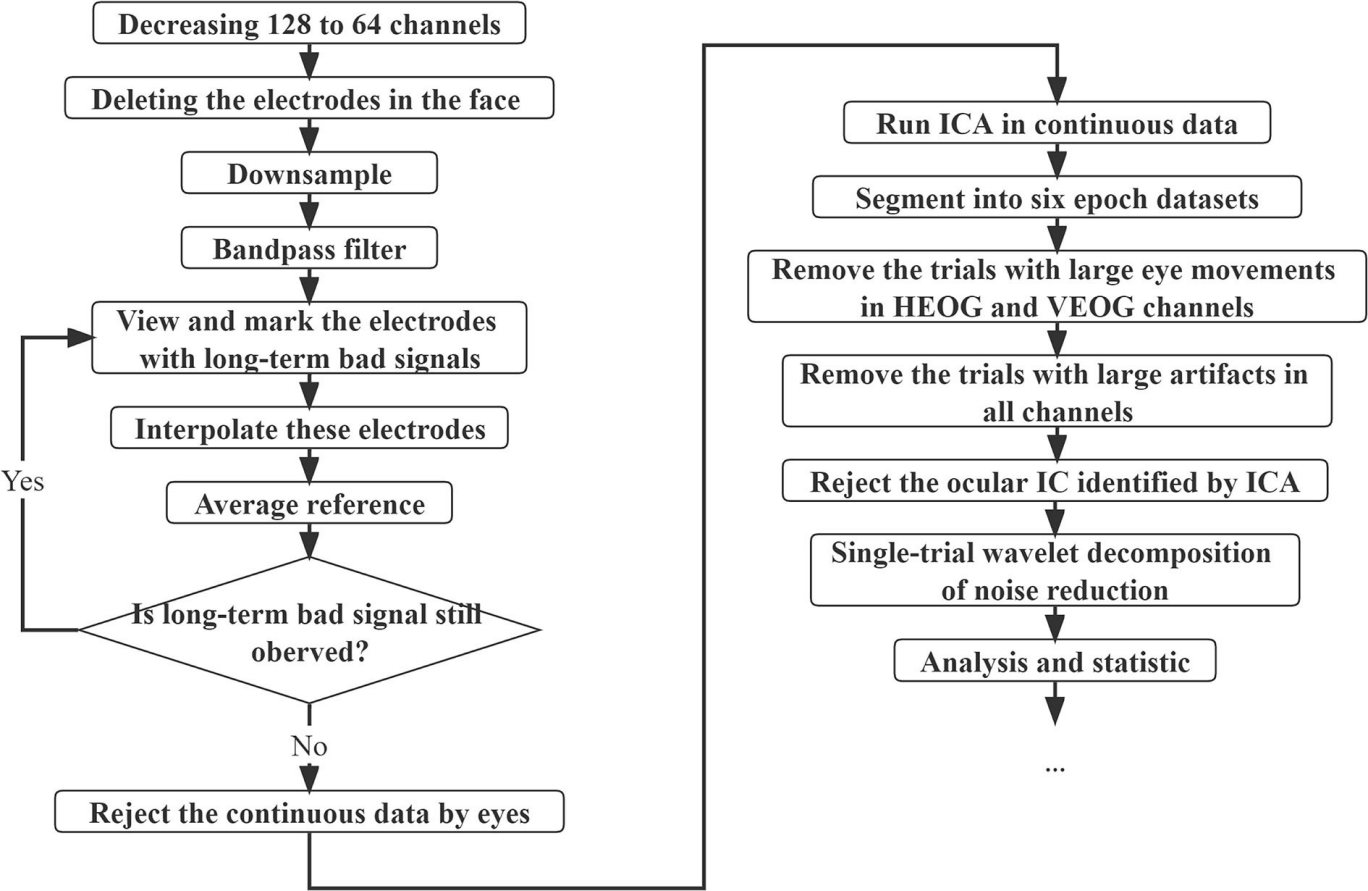
Flowchart of the entire pre-processing procedure. The data were decreased from 128 to 64 channels if collected with the 128-channel system. Then, four electrodes in the face were deleted. The data were downsampled at 250 Hz, and the bandpass was filtered at 1–30 Hz. After interpolating all the electrodes with long-term bad signals, the data were average re-referenced. After visually inspected to remove artifacts, the independent component analysis (ICA) was conducted on the continuous EEG data. Then, the continuous time series were segmented into six epoch datasets from −200 to 400 ms relative to stimulus onset with baseline correction (−200 to 0 ms). The trials with large eye movements and large artifacts were removed. For the remaining trials, the ocular ICs identified by ICA were rejected, and then single-trial wavelet decomposition was used to reduce noise.

Furthermore, offline pre-processing of the data was conducted using custom scripts with the function from the EEGLAB (Delorme and Makeig, 2004) in MATLAB (The Mathworks, Inc., Natick, MA, USA), and the flowchart of the entire pre-processing procedure is presented in Figure 2. For data collected with the 128-channel system, the data of homologous channels were obtained when decreasing from 128 to 64 channels, and for all data, four electrodes in the face (Nos. 61, 62, 63, and 64 in the 64-channel EGI system) were deleted. The data were downsampled at 250 Hz and then bandpass filtered at 1–30 Hz with the default FIR filter in EEGLAB. After interpolating all the electrodes with long-term bad signals, the data were re-referenced with the average of the signal at all EEG electrodes. The continuous EEG data were visually inspected to remove irregular amplifier saturation or movement artifacts. Next, the independent component analysis (ICA) was conducted with the function “runica” and the extended option of “pca” (20 components) in the EEGLAB (Jung et al., 2000; Delorme and Makeig, 2004) with the extended infomax method (Lee et al., 1999). To identify the ocular artifacts better, the principal component analysis (PCA) algorithm with 20 reduction dimensions was suitable as a precursor to ICA (Lee et al., 1999; Artoni et al., 2018) to reduce the dimension of redundant ICA components. Then, the continuous time series were segmented into six epoch datasets from −200 to 400 ms relative to stimulus onset with baseline correction (−200 to 0 ms).

According to a previous study, there was a clinical linkage between the alpha rhythm and the eye blink rate (Sadato et al., 1998). Meanwhile, eye movement has a function linked with alpha modulation (van Zoest et al., 2021; Liu et al., 2022). These studies suggest that ocular artifacts during the presentation of a stimulus would confuse the group difference just by simply removing these ICA weights. In particular, it is more difficult for children to control eye blinking and eye movement compared with adults. To address this, we first (1) ran ICA to identify the weights of eye blinking and eye movement and then (2) rejected trials with large horizontal eye movements from 200 to 350 ms. To further remove the horizontal eye movements in the data, we rejected the trials with the difference of electrodes F9/10 (No. 17/1 in 64-channel EGI system) exceeding ±50 μV during the entire trials. In addition, to further remove the vertical eye movements during the presentation of the stimuli, we also rejected the trials with the mean waves of electrodes FP1/2 (No. 10/5 in 64-channel EGI system) exceeding ±75 μV during the entire trials. The trials with absolute voltage values exceeding 100 μV in any channel were rejected. Finally, (3) we removed identified ICA weights of ocular artifacts. To assess whether any systematic horizontal EOG activity was present in the remaining data, we computed averaged HEOG waveforms for left and right target trials. Residual activity was < 2 μV (see Supplementary Table S1), which showed that the residual eye movements were < ±0.3° (McDonald and Ward, 1999). Thus, the small horizontal eye movements suggested that participants kept their eyes fixating on the center of the screen.

A single-trial wavelet decomposition (a linear increase from 3 cycles to 30 cycles in 1–30 Hz) of noise reduction (retaining 90% of main signals) was used to enhance the signal-to-noise ratio (SNR) (Hu et al., 2010, 2011), which used the same time-frequency resolution as in the previous literature (Iannetti et al., 2008).

#### ERP data

Only the complete trials that did not contain artifacts and incorrect responses were further analyzed. Participants would be excluded from the further analysis if they had an insufficient number of trials (<70%). There were no differences in the residual trial numbers of the three types of stimuli between groups (ADHD group: target stimuli: 210 ± 35; non-target stimuli: 216 ± 38; standard stimuli: 883 ± 160, TD group: target stimuli: 219 ± 17; non-target stimuli: 225 ± 16; standard stimuli: 923 ± 72, *p*s > 0.065). According to the spatial location of the deviant stimulus presented, four data bins were collected (target stimuli on the left side, target stimuli on the right side, non-target stimuli on the left side, and non-target stimuli on the right side) for each subject in four pairs of symmetrical electrodes (No. 15/53, No. 22/49, No.26/46, No.28/42 in 64-channel EGI system) between parietal lobe and temporal lobe (Gamble and Luck, 2011; Gamble and Woldorff, 2015b). The lateralization effect was investigated by measuring the mean amplitude over consecutive 50-ms time intervals from 200 ms with a permutation test corrected within groups (Gamble and Luck, 2011; Gamble and Woldorff, 2015b; Klatt et al., 2018).

**Figure 3:**
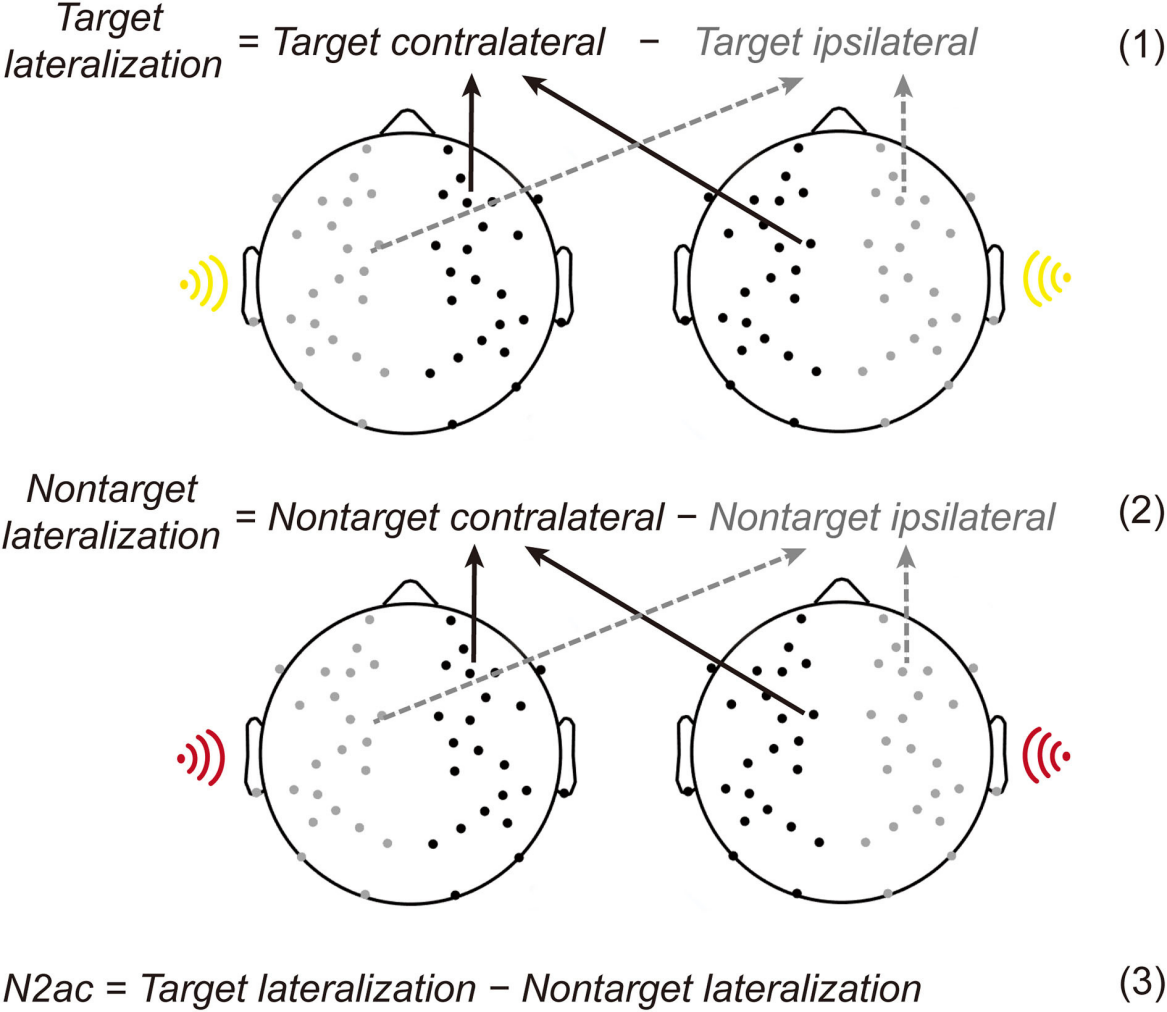
Illustration of lateralized ERP components. The target/non-target deviants of ipsilateral voltage were the average of the right deviants elicited voltage in the right hemisphere and the left deviants elicited voltage in the left hemisphere. And the target/non-target deviants of contralateral voltage were the average of the right deviants elicited voltage in the left hemisphere and the left deviants elicited voltage in the right hemisphere. Then, the lateralization effect was the difference in subtracting the ipsilateral voltage from the contralateral voltage [see the Formulas (1) and (2)]. The N2ac component reflecting auditory spatial selective attention was calculated by subtracting the lateralization in non-target deviants from the target deviants [see the Formula (3)].

In the current task, the contralateral-minus-ipsilateral difference wave of target deviants or non-target deviants was calculated using the four data bins (Figure 3). The target/non-target deviants of ipsilateral voltage were the average of the right deviants elicited voltage in the right hemisphere and the left deviants elicited voltage in the left hemisphere. The target/non-target deviants of contralateral voltage were the average of the right deviants elicited voltage in the left hemisphere and the left deviants elicited voltage in the right hemisphere. Then, the lateralization effect was the difference in subtracting the ipsilateral voltage from the contralateral voltage. Similar to the typical N2pc in the visual domain, the lateralized spatial attention ERP component is described in the formulas (1) and (2) in Figure 3.

According to previous studies, the N2ac component reflecting auditory spatial selective attention was calculated by subtracting the lateralization in non-target deviants from the target deviants (see the formula (3) in Figure 3; Gamble and Woldorff, 2015b).

### Statistical analysis

All statistical analyses were performed using SPSS 26.0 (IBM Corporation, Somers, NY). For behavioral and clinical data, two-tailed independent sample *t-test*s were conducted on RT, RT_CV_, ERROR, and ADHD symptom scores between the two groups. For EEG data, two-tailed paired sample *t-*tests were conducted first on ERP waveforms of (non)target deviants (contralateral vs. ipsilateral) to test the lateralization effect for both groups (ADHD group and TD group); moreover, based on the lateralization effect, two-tailed 2 × 2 ANOVA (Group: ADHD group and TD group; Condition: target and non-target) was conducted to test the N2ac component, followed by *post-hoc* comparison with Bonferroni correction. Considering that non-normal distribution had been observed in the groups with deficits (Tarantino et al., 2013; Lin et al., 2014; Lee et al., 2015; Hwang-Gu et al., 2019), the Mann–Whitney–Wilcoxon tests were also conducted in behavioral and EEG data to confirm the results from parametric statistics. For the relationship between behavioral data and EEG data, Pearson correlations were conducted between behavioral/clinical data (RT, RT_CV_, ERROR, and ADHD symptom scores) and EEG data (the lateralization effect and the N2ac component) for each group with age and sex controlled. The significance of the correlation was corrected for multiple comparisons using the false discovery rate (FDR) method.

## Results

### Behavioral, demographic, and clinical results

**Figure 4:**
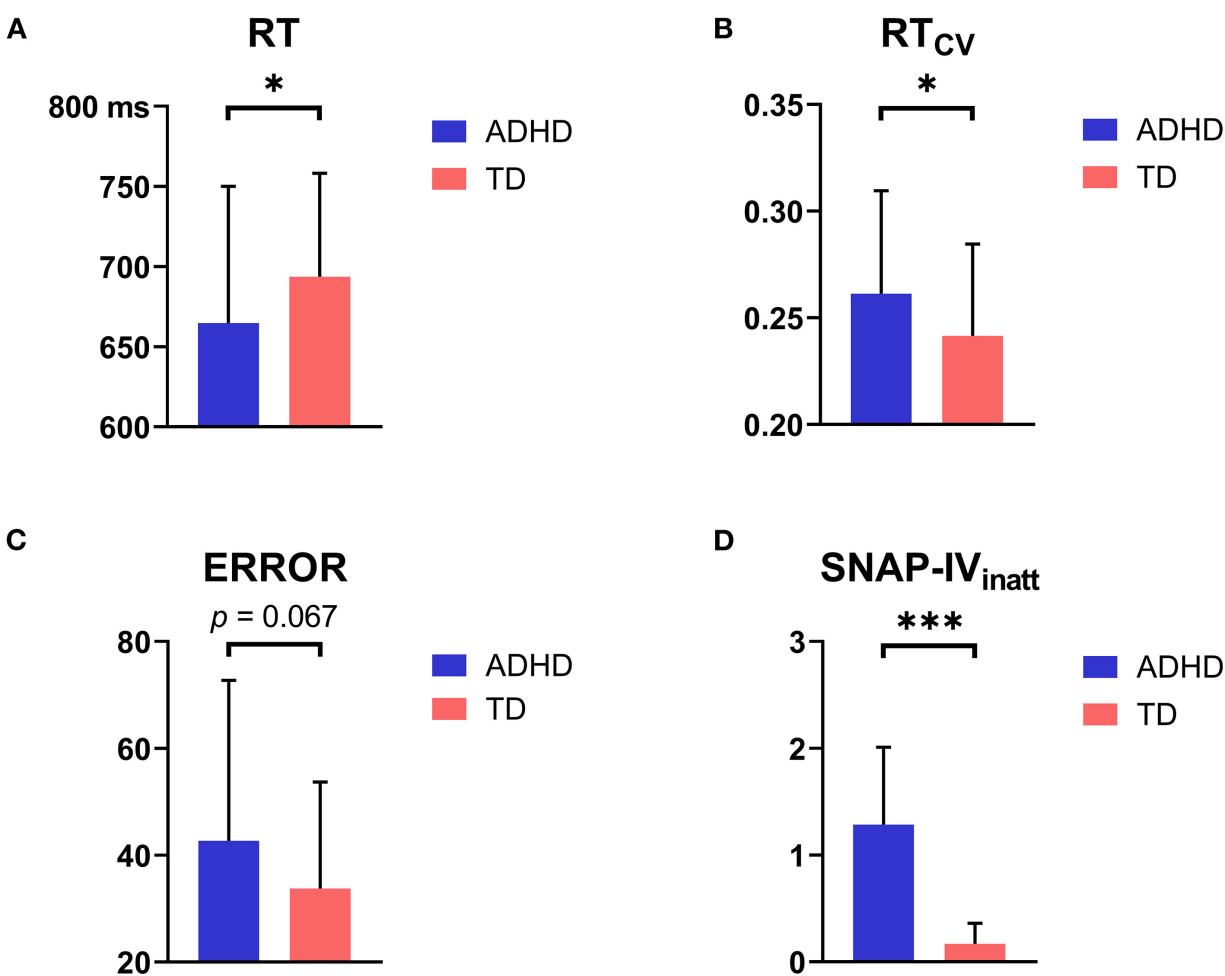
Group differences in behavioral and clinical results. **(A)** Children with ADHD had significantly shorter RT than TD children. **(B)** Children with ADHD had significantly higher RT_CV_ than TD children. **(C)** Children with ADHD had marginally higher ERROR than TD children. **(D)** Children with ADHD had significantly more severe ADHD symptoms than TD children (represented by SNAP-IV_inatt_ Score). *p < 0.05; ***p < 0.001.

Group comparisons of behavioral performance are shown in Table 2 and Figure 4. Children with ADHD had significantly shorter RT (*t* = −2.060, *p* = 0.042) and higher RT_CV_ (*t* = 2.332, *p* = 0.021) than TD children. A marginally higher ERROR was observed in the children with ADHD than in the TD children (*t* = 1.902, *p* = 0.060). Moreover, children with ADHD had significantly more severe ADHD symptoms (*ps* < 0.001, within SNAP-IV_inatt_, SNAP-IV_hyper_, and SNAP-IV_full_) than TD children.

**Table 2:** Group differences in behavioral performance and ERP measures in the auditory selective attention task.

	**ADHD (*n* = 54)**	**TD (*n* = 61)**	***t***	***df***	***p***
**Behavior (mean** **±SD)**					
RT	664.82 ± 85.31	693.67 ± 64.47	−2.026	98	0.046
RT_CV_	0.26 ± 0.05	0.242 ± 0.043	2.332	113	0.021
ERROR	42.72 ± 30.00	33.79 ± 19.89	1.857	90	0.067
**ERP measures (mean** **±SD)**					
Target lateralization	−0.38 ± 0.27	−0.40 ± 0.26	0.279	113	0.781
Non-target lateralization	−0.34 ± 0.20	−0.22 ± 0.25	−2.899	112	0.005
N2ac component	−0.04 ± 0.28	−0.18 ± 0.31	2.468	113	0.015

ADHD, children with attention-deficit/hyperactivity disorder; TD, TD children; RT, reaction time; RT_*CV*_, RT coefficient of variability, reflected the variability and deviation in response, calculated from the standard deviation of RT; ERROR, the individual total number of error response; Target lateralization, target deviants elicited lateralization effect in 200–350 ms; Non-target lateralization, non-target deviants elicited lateralization effect in 200–350 ms; N2ac, subtracting non-target deviants elicited lateralization effect from target deviants elicited lateralization effect in 200–350 ms; SD, standard deviation.

### Lateralization effect

**Figure 5:**
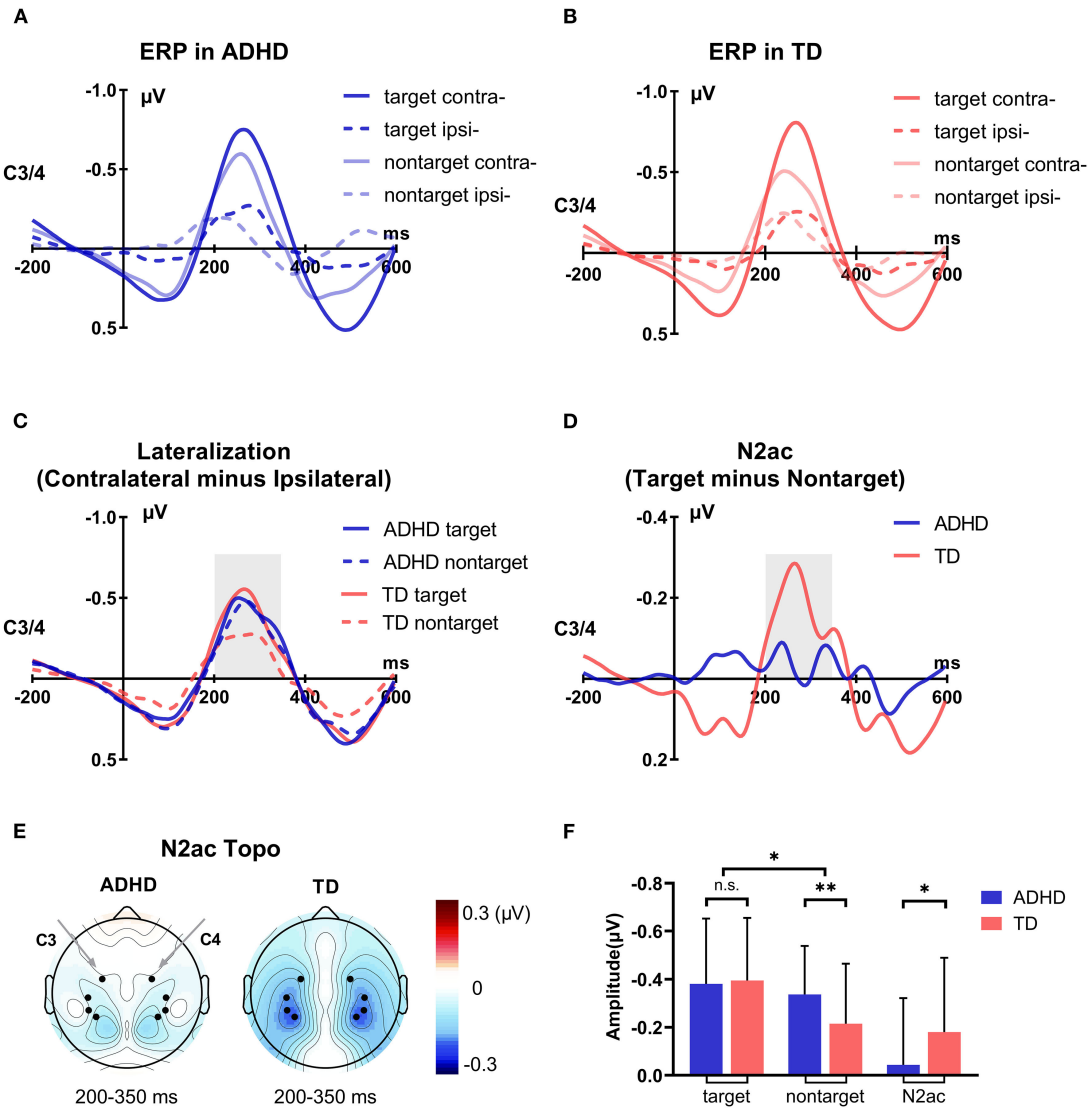
Group difference in electrophysiology results. **(A)** The grand-average ERPs at contralateral and ipsilateral electrode sites to the target deviants and non-target deviants of the ADHD group. **(B)** The grand-average ERPs at contralateral and ipsilateral electrode sites to the target deviants and non-target deviants of the TD group. **(C)** The grand-average contralateral-minus-ipsilateral difference waveforms of the (non)target deviants in the two groups, namely, the lateralization effect. **(D)** The grand-average target-minus-non-target difference waveforms of the lateralization effect, namely, the N2ac component. **(E)** The corresponding topographic maps of the N2ac component of the two groups. **(F)** Statistical analysis results of the ERP data (target lateralization, non-target lateralization, and N2ac component). *p < 0.05; **p < 0.01.

The ERP waveforms in response to the auditory stimuli from electrodes over the temporal cortex contralateral and ipsilateral to the target and non-target are shown in the ADHD group (Figure 5A) and TD group (Figure 5B). According to previous studies in adults (Gamble and Luck, 2011; Gamble and Woldorff, 2015b; Klatt et al., 2018, 2020), the lateralization effect was stably observed beginning ~200 ms from auditory stimulus onset; thus, the lateralization effect was investigated by measuring the mean amplitude over consecutive 50-ms time intervals from 200 ms with a permutation test corrected within groups. Paired sample *t-*tests showed that ERP waveforms at the contralateral site were significantly more negative than those at the ipsilateral site during 200–350 ms for the target/non-target deviants in both groups (*p*s < 0.001). The lateralization effect was also seen as a negative deflection during 200–350 ms after auditory deviant onset in the contralateral-minus-ipsilateral difference waveforms in the parietal lobe and temporal lobe in both groups (Figure 5C). Moreover, it seemed that the amplitudes of contralateral-minus-ipsilateral difference waveforms were comparable during 200–350 ms among these conditions, except that the lateralization effect elicited by non-target deviants in TD children was weaker than that in other children.

### The N2ac component

To assess the statistical significance of these lateralization effects between the two groups, based on the significant time period of the lateralization effect, the mean ERP voltage in contralateral-minus-ipsilateral difference waveforms was measured using ANOVA from 200 to 350 ms after auditory stimulus onset. The results showed that the significant main effect of Condition [*F*_(1, 113)_ = 16.458, *p* < 0.001, $\eta_{p}^{2}$ = 0.127] and the interaction of Condition × Group [*F*_(1, 113)_ = 6.093, *p* = 0.015, $\eta_{p}^{2}$ = 0.051] was observed in ANOVA, which confirmed that the N2ac component was different between the two groups. *Post-hoc* comparison with Bonferroni correction showed a significant difference between target and non-target deviants in TD children (*p* < 0.001) but not in children with ADHD (*p* = 0.278). For a more intuitive comparison, we subtracted the different ERP waveforms of non-target deviants from the different ERP waveforms of target deviants. The N2ac component was observed at 200–350 ms in the parietal lobe and temporal lobe in the TD group but not in children with ADHD (Figures 5D,E). For a further group comparison, the N2ac component in children with ADHD was significantly smaller than that in TD children (*t* = 2.468, *p* = 0.015; Figure 5F). The results showed that the N2ac component was absent in children with ADHD due to their similar lateralization to the target and the non-target deviant sounds.

The results of Mann–Whitney–Wilcoxon tests in behavioral and EEG data were similar to those of the parametric test (ERROR: *z* = −1.256, *p* = 0.209; non-target lateralization: *z* = −2.455, *p* = 0.014; RT: *z* = −1.544, *p* = 0.123; RT_CV_: *z* = −2.059, *p* = 0.039; target lateralization: *z* = −0.566, *p* = 0.571; N2ac: *z* = −2.113, *p* = 0.035). Considering that the sample size was more than 40 for each group, the non-parametric analyses suggested that the results of the parametric tests were sufficiently acceptable.

### Relationships between behavior, symptoms, and ERP measures

**Figure 6:**
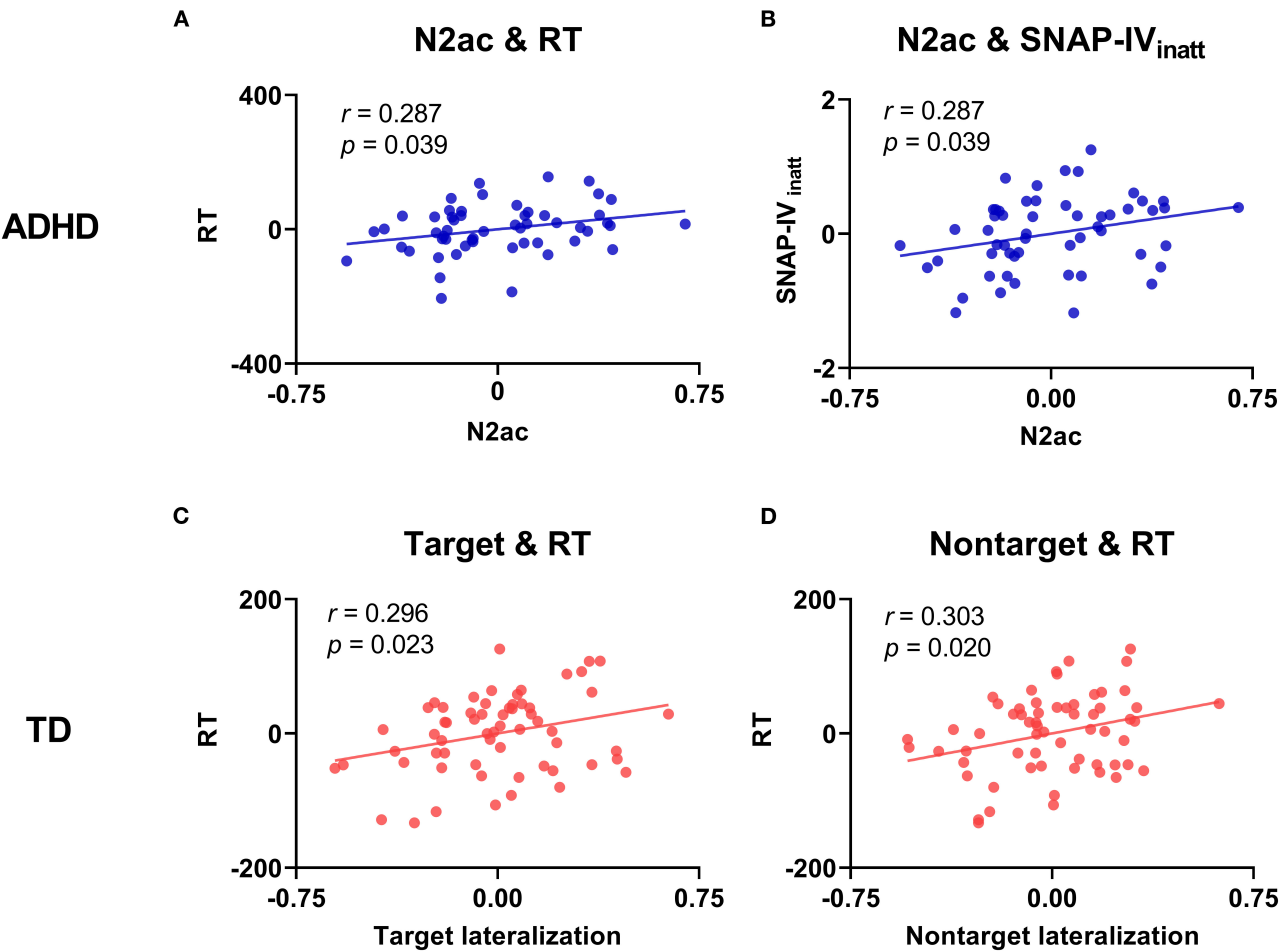
Scatter plots between the behavioral/clinical data (RT, ERROR, and ADHD symptom score) and EEG data (target lateralization, non-target lateralization, and N2ac component). **(A)** A significant correlation between the N2ac component and RT in the ADHD group. **(B)** A significant correlation between the N2ac component and inattention symptom score (SNAP-IV_inatt_) in the ADHD group. **(C)** A significant correlation between target lateralization and RT in the TD group. **(D)** A significant correlation between non-target lateralization and RT in the TD group.

For each group, we constructed a correlation analysis (with FDR corrected) between the behavioral performance (RT, RT_CV_, ERROR, and ADHD symptoms) and ERP measures (the lateralization effect and the N2ac component) (see Figure 6 and Supplementary Tables S3–S5). The results showed that the larger N2ac component was significantly correlated with faster RT (*r* = 0.287, *p* = 0.039) and milder inattention symptom scores (SNAP-IV_inatt_) (*r* = 0.287, *p* = 0.039) for children with ADHD. On the other hand, the larger target and non-target lateralizations were significantly correlated with faster RT (target: *r* = 0.296, *p* = 0.023; non-target: *r* = 0.303, *p* = 0.020) for TD children. No other significant correlation was found. The results showed that a larger N2ac component was associated with better behavioral performance and milder inattention symptom scores in the ADHD group. However, the larger lateralization of target and non-target was associated separately with better behavioral performance in the TD group.

## Discussion

People often need to focus on the sound of interest and ignore other irrelevant sounds in real auditory circumstances, in which the ability of auditory spatial selective attention plays an important role. The current study investigated this ability in children. The results showed that children with ADHD had impulsive behavioral performance (higher RT_CV_, shorter RT, and marginally higher ERROR) and a dysfunctional N2ac component compared to TD children, which originated from the smaller non-target lateralization effect in TD children. Further correlation analyses showed that the higher N2ac component in children with ADHD was accompanied by faster RT and milder ADHD symptoms.

Behaviorally, we found that children with ADHD had higher RT_CV_, faster RT, and marginally higher ERROR than TD children. According to previous studies, the higher RT_CV_ and marginally higher ERROR in children with ADHD reflected their characteristics of deficits in inhibition (Smith et al., 2004; Rommelse et al., 2007; Tamm et al., 2012; Kofler et al., 2013). In addition, there was a low response probability of target stimulus (16.3%) and a high possibility of no response in this oddball paradigm (83.7%). Although the target was easy to identify, the unpredictable inter-target interval made a scene of “waiting” for children with ADHD who suffered from the characteristics of delay aversion and dysfunctional impulsivity (Overtoom et al., 1998). As a result, children with ADHD performed at risk with shorter RT, which indicated deficits in executive control as described in dual pathway models of ADHD (Sonuga-Barke, 2003). However, the slower RT in TD children might be due to more cognitive resources that they invested in balancing the attention allocation in target and non-target deviants; thus, they balanced the speed-accuracy trade-off, which was confirmed by further correlation analyses (please see below). Therefore, these behavioral results indicated impairment in regulating the speed-accuracy trade-off in children with ADHD, as in previous research (Mulder et al., 2010).

According to a previous study, the reaction times of left/right sound intensity were different in normal and AD/HD children (Baghdadi et al., 2017). We also confirmed the behavioral performance and ERP components with the left and right sides of sounds separately (see Supplementary Figure S1), and there were no significant interactive effects with the two groups (*ps* > 0.440), suggesting that the results in our study were not confused with the difference in left/right sounds. Using contralateral-minus-ipsilateral difference waveforms, the lateralization effect of deviants counteracted the effects of physical stimulation asymmetry, reflecting automatic attention shifting to unilateral salient sounds (Gamble and Luck, 2011; Hillyard et al., 2016); meanwhile, the pseudorandom presentation of stimuli had also balanced the influence of the difference in left/right sounds.

In the current study, the lateralization effect was the negative deflection during 200–350 ms after the onset of auditory deviants in the parietal lobe and temporal lobe in both groups (Figure 5C), which was similar to the research in adults (Gamble and Luck, 2011; Gamble and Woldorff, 2015b; Lewald and Getzmann, 2015; Klatt et al., 2018; Getzmann et al., 2020), suggesting that children could allocate selective spatial attention to unilateral deviants, whether the deviants were target or non-target. Further correlation analyses showed that the higher lateralization effects of deviants were directly accompanied by faster RT only in TD children, consistent with previous studies showing that the high ability of automatic attention allocation to deviants could accelerate behavioral performance (Gamble and Luck, 2011; Hillyard et al., 2016). Although additive non-target suppression consumed more time, TD children had lower RT_CV_ and less ERROR, which suggested that TD children invested more cognitive resources in balancing the processes of focusing the target and suppressing the non-target, implying a strategy of a speed-accuracy trade-off. Therefore, this additive suppression process contributed to their robust N2ac, similar to adults (Gamble and Luck, 2011; Gamble and Woldorff, 2015b; Klatt et al., 2018, 2020).

The lateralization effect showed that children could allocate selective spatial attention to unilateral deviants; however, after subtracting non-target lateralization from target lateralization, the robust N2ac component was only observed in TD children, which resulted from a group difference in the non-target lateralization effect. The smaller non-target lateralization effect was observed only in TD children, while there was no group difference in the target lateralization effect. These results indicated that only TD children could efficiently inhibit the automated processing of irrelevant deviants, and the robust N2ac component reflected that they had developed an “adult-like” ability to balance auditory selective attention and distractor suppression (Gamble and Luck, 2011; Gamble and Woldorff, 2015b; Klatt et al., 2018, 2020). However, children with ADHD did not develop this inhibition ability with delay aversion, as described in the dual pathway model (Sonuga-Barke, 2003; Halperin et al., 2008), and thus had a deficiency in auditory selective attention in electrophysiological activity, although the lateralization effect of deviants could be observed.

Furthermore, the current study showed that the N2ac might be associated with the auditory spatial selective attention process in the “cocktail-party effect.” An involuntary attention three-stage model described this process as the “cocktail-party effect” (Escera and Corral, 2007; Wetzel and Schröger, 2014). First, unpredictable audio will be detected automatically (observing typically MMN); subsequently, involuntary attention will be abstracted by the unpredictable audio; finally, selective attention will emerge, directing task-related information with attention extracted from irrelevant deviants. The N2ac component in children with ADHD indicated their deficiency of auditory spatial selective attention in the latter two processes of the model, which is consistent with previous studies focusing on the two aspects separately: (1) inhibiting automatic attentional shift (Smith et al., 2004) and/or (2) inefficient attentional allocation (Sawaki and Katayama, 2006). Therefore, compared to the dysfunctional non-target lateralization effect, the dysfunctional N2ac component in children with ADHD provided novel evidence for the involuntary attention three-stage model with the development of auditory selective attention.

The most novel and critical point from our study are that the N2ac component could be observed in children, where the N2ac absent in children with ADHD suggested the association with poorer behavioral performance/higher inattentive symptom severity. First, considering the previous research in adults, our findings indicated that 7- to 11-year-old TD children have developed “adult-like” N2ac, suggesting that the ability of TD children to balance auditory selective attention and distractor suppression was similar to that of adults (Gamble and Luck, 2011; Gamble and Woldorff, 2015b; Klatt et al., 2018, 2020); meanwhile, the N2ac component of TD children, observed at the anterior contralateral electrode sites 200 to 350 ms after the stimulus onset in the N2 latency in our study, was consistent with previous studies in the lateral prefrontal cortex (LPFC), which suggested the association with the control of auditory attention (Bidet-Caulet et al., 2015). Second, considering previous debates on the auditory-related EEG components such as MMN or Nd in patients with ADHD (Satterfield et al., 1988; Jonkman et al., 1997; Rothenberger et al., 2000; Huttunen et al., 2007; Itagaki et al., 2011; Gomes et al., 2012; Cheng et al., 2016; Yamamuro et al., 2016; Rydkjær et al., 2017; Zhang et al., 2020; le Sommer et al., 2021), our findings provide the first evidence for the difference in the spatial auditory selective attention between children with ADHD and TD children, where the N2ac was absent in children with ADHD. Third, we found that the dysfunctional N2ac of children with ADHD was associated with higher inattentive symptom severity, such as the function of N2pc in visual attention (Wang et al., 2016).

There are also some limitations. First, larger samples would be needed to validate our results and explore the potential clinical value, such as aiding diagnosis and designing targeted training. Moreover, there are three subtypes of ADHD: ADHD-inattention type, ADHD-impulsive/hyperactive type, and ADHD-combined type. Due to the limited size of the samples, we did not differentiate among the subtypes or further subgroups of ages. Second, compared with other auditory studies, there may be some other limitations on the application of the N2ac component. For example, patients must focus their attention on the auditory stimuli throughout the task, where the elicited N2ac was intrinsically different from the MMN component of the inattentive situation. Besides, as both visual N2pc/P_D_ and auditory N2ac were associated with the severity of symptoms, we would investigate the contributions and the relationships between the selective attention of the two modalities in further studies on ADHD.

## Conclusion

Our auditory spatial selective attention task provides novel and robust neurobiological evidence that attention problems in ADHD are at least partially related to poor auditory spatial selective attention (as reflected by the N2ac component). Our findings provide a neurophysiologic basis for understanding attention deficits in children with ADHD and highlight the importance of auditory spatial attention in ADHD.

## Data availability statement

The datasets generated during the current study are available from the corresponding authors upon reasonable request.

## Ethics statement

The studies involving human participants were reviewed and approved by the Affiliated Brain Hospital of Guangzhou Medical University. Written informed consent to participate in this study was provided by the participants' legal guardian/next of kin. Written informed consent was obtained from the minor(s)' legal guardian/next of kin for the publication of any potentially identifiable images or data included in this article.

## Author contributions

CD, YS, and LC designed the experiment. TF, BL, SH, and HL performed the experiment. TF and BL analyzed the data. WY, XL, and HS helped to recruit the children. WY, YZ, and DC diagnosed the children. TF, BL, YS, and CD wrote the manuscript. All authors reviewed and approved the final manuscript.
