# Supplementary material for: Sound localization and auditory selective attention in school-aged children with ADHD
Source: Front Neurosci. 2022 Dec 15;16:1051585. doi: 10.3389/fnins.2022.1051585 (PMC9812578; doi:10.3389/fnins.2022.1051585)
Supplement: Supplementary file 1 [file Presentation_1.pdf]

Supplementary Material

1 Ta

When processing lateralization of auditory deviants, a positive component, Ta, was observed in the current study, which was a bilateral component considered to be related to early perceptual processing over the temporal cortex, as in previous studies (Gomes et al., 2012; Groen et al., 2008; Tonnquist-Uhlen et al., 2003; Ponton et al., 2002; Wolpaw and Penry, 1975). The lateralized Ta effect was elicited by both the target and nontarget deviants at approximately 100 ms for both groups (**Figure 5C**) with contralateral-minus-ipsilateral difference waveform, which was similar to previous studies (Tonnquist-Uhlen et al., 2003; Tonnquist-Uhlén, 1996; Wolpaw and Penry, 1975). The lateralized Ta effect occurred prior to the N2ac effect, which was observed in the lateralization effect of the target and nontarget deviants at 200−350 ms for both groups. One-sample *t tests* revealed that the amplitude of contralateral-minus-ipsilateral Ta was significantly different from zero for target and nontarget deviants of both groups ($ps < 0.001$). However, it seemed that the lateralized Ta effect had comparable amplitudes during 50−150 ms among target and nontarget deviants of both groups except that the smaller lateralized Ta effect elicited by nontarget deviants in TD children (paired sample *t tests*: ADHD: $t = -0.896$, $p = 0.374$; TD: $t = 3.068$, $p = 0.003$).

To investigate the lateralized Ta effect, a Condition (target deviants, nontarget deviants) × Group (ADHD, TD) ANOVA was conducted. Only a significant interaction of Condition × Group ($F_{(1,113)} = 6.510$, $p = 0.012$, $\eta_p^2 = 0.054$) was observed in ANOVA, which confirmed that the Ta component was different between the two groups. Post hoc comparison with correction showed that only TD children had a significant difference in lateralized Ta effect between target deviants and nontarget deviants ($F = 6.975$, $p = 0.009$, $\eta_p^2 = 0.058$), and a significant group difference in nontarget deviants elicited lateralized Ta effect ($F = 6.803$, $p = 0.010$, $\eta_p^2 = 0.057$), which led the higher target-minus-nontarget lateralized Ta effect of TD children than that of children with ADHD ($t = -2.552$, $p = 0.012$, seen in **Table S2**). For a more intuitive comparison, we subtracted the difference ERP waveform of nontarget deviants from the difference ERP waveform of target deviants, similar to the formula of N2ac (seen in **Figure 5D**). The results showed the group difference in early stimuli processing of target deviants and nontarget deviants.

Because the Ta component reflected early basic stimulus processing, we explored the relationship between Ta and behavior performance. In this study, no significant correlation was found between the lateralized Ta effect and behavior performance for either group ($ps > 0.128$), which indicated that Ta was not related to the current auditory search task (seen in **Table S3** and **Table S4**).

In the early stage of ERP induced by deviants, we also found that children with ADHD had abnormal perceptual processing in stimulus features compared with TD children. Comparing target deviants with nontarget deviants, target deviants induced Ta was significantly larger than nontarget deviants induced Ta in the TD group, suggesting that TD children had the ability of efficient attentional allocation of initial perceptual process between the target deviants and the nontarget deviants. However, no difference in target/nontarget deviants induced Ta in the ADHD children, suggesting that children with ADHD might lack the ability to inefficiently allocate attention to nontarget

deviants in the initial perceptual process, which was consistent with the view of inhibitory deficits in executive control in the dual pathway models of ADHD (Sonuga-Barke, 2003).

2 Analysis of group differences between left and right stimuli

We divided the stimuli into left and right data for each subject, and used ANOVA (Group: ADHD, TD; Stimulus Side: left, right; Condition: Target, Nontarget) to compare presentation difference in ERP and behavioral results within different groups. For RT, we found the significant main effect of Stimulus Side ($F_{(1,113)} = 4.410$, $p = 0.038$, $\eta_p^2 = 0.038$), but no interaction effect ($F_{(1,113)} = 0.600$, $p = 0.440$, $\eta_p^2 = 0.005$). Post hoc comparison with Bonferroni correction showed that TD children had slower RT when the sound played on the left than right ($p = 0.038$), but not in children with ADHD. The behavioral results suggested that there was a difference in RT when sound was played in the right or left ear between the two groups, which coincided with the ideas in Baghdadi et al. (2017). However, different from the results in Baghdadi et al. (2017), we found that the difference in left-/right- stimuli orientation was only observed in TD children. This might result from the following factors: (1) The way of response was different. We restricted keystrokes only to the right hand (handedness), while "the subjects were requested to touch a button as soon as hearing a beep sound" in Baghdadi et al. (2017). Whether the difference in using left or right hand between TD children and children with ADHD remains to be investigated. (2) The task was different. Only target stimuli (16.3% occurrence) were required to respond to in our study, which involves both attention (Go) and inhibition (NoGo). However, Baghdadi et al. focused on the sensitivity of the left and right pathways to different sound intensities. In their study, the subjects were requested to touch the button as soon as possible when hearing any auditory stimuli in any ear, which did not involve inhibition (NoGo). The results of RT_{CV} and ERROR were not statistically significant.

For ERP data, we calculated the average amplitude of each subject at different sides for different stimuli, and the ranges of time and electrodes were consistent with the analysis of N2ac (time: 200–350 ms; electrodes: No.15/53, No.22/49, No.26/46, No.28/42 in 64-channel EGI system). There was no main effect of Stimulus Side ($F_{(1,113)} = 1.885$, $p = 0.173$, $\eta_p^2 = 0.016$), and no effect of Group × Stimulus Side × Condition ($F_{(1,113)} = 0.149$, $p = 0.700$, $\eta_p^2 = 0.001$), suggesting no difference between the two groups. Post hoc tests revealed that the difference was mainly in Condition (Target, Nontarget; seen in **Figure S1**), and the results were consistent with the analysis of N2ac.

3 References

Baghdadi, G., Towhidkhah, F., and Rostami, R. (2017). Left and right reaction time differences to the sound intensity in normal and AD/HD children. *Int J Pediatr Otorhinolaryngol* 97, 240–244. doi: 10.1016/j.ijporl.2017.04.025.

Gomes, H., Duff, M., Ramos, M., Molholm, S., Foxe, J. J., and Halperin, J. (2012). Auditory selective attention and processing in children with attention-deficit/hyperactivity disorder. *Clinical Neurophysiology* 123, 293–302. doi: 10.1016/j.clinph.2011.07.030.

Groen, M. A., Alku, P., and Bishop, D. V. M. (2008). Lateralisation of auditory processing in Down syndrome: a study of T-complex peaks Ta and Tb. *Biol Psychol* 79, 148–157. doi: 10.1016/j.biopsycho.2008.04.003.

Ponton, C., Eggermont, J. J., Khosla, D., Kwong, B., and Don, M. (2002). Maturation of human central auditory system activity: separating auditory evoked potentials by dipole source modeling. *Clin Neurophysiol* 113, 407–420. doi: 10.1016/s1388-2457(01)00733-7.

Sonuga-Barke, E. J. S. (2003). The dual pathway model of AD/HD: an elaboration of neuro-developmental characteristics. *Neurosci Biobehav Rev* 27, 593–604. doi: 10.1016/j.neubiorev.2003.08.005.

Tonnquist-Uhlén, I. (1996). Topography of auditory evoked long-latency potentials in children with severe language impairment: the T complex. *Acta Otolaryngol* 116, 680–689. doi: 10.3109/00016489609137907.

Tonnquist-Uhlen, I., Ponton, C. W., Eggermont, J. J., Kwong, B., and Don, M. (2003). Maturation of human central auditory system activity: the T-complex. *Clin Neurophysiol* 114, 685–701. doi: 10.1016/s1388-2457(03)00005-1.

Wolpaw, J. R., and Penry, J. K. (1975). A temporal component of the auditory evoked response. *Electroencephalography and Clinical Neurophysiology* 39, 609–620. doi: 10.1016/0013-4694(75)90073-5.

Table S1. The mean amplitudes of HEOG before and after removing ocular artifacts (Mean±SD, μV).

	0−200 ms	200−350 ms
Before HEOG	0.02±0.71	0.40±2.79
After HEOG	0.00±0.00	0.00±0.00

Table S2. Group difference in the lateralized Ta effect.

	ADHD (n = 54)	TD (n = 61)	t
**Lateralized Ta (Mean±SD)**			
Target Ta	0.22 ± 0.30	0.26 ± 0.27	−0.844
Nontarget Ta	0.27 ± 0.25	0.14 ± 0.28	2.608*
Target-minus-nontarget Ta	−0.05 ± 0.41	0.12 ± 0.31	−2.552*

ADHD, children with attention-deficit/hyperactivity disorder; TD, typically developmental children; Target Ta, target deviants elicited lateralized Ta effect; Nontarget Ta, nontarget deviants elicited lateralized Ta effect; Target-minus-nontarget Ta, subtracting nontarget deviants from target deviants in lateralized Ta effect; Mean, average value; SD, standard deviation; *significance with correct (p value) < 0.05.

Table S3. The correlation between two types of ERP lateralization and behavior performance in children with ADHD (*r* value)

	RT	RT_{CV}	ERROR
50−150 ms			
Target Ta	−0.199	0.154	0.122
Nontarget Ta	0.069	−0.007	−0.040
Target-minus-nontarget Ta	−0.184	0.115	0.111
200−350 ms			
Target lateralization	0.250	0.212	0.154
Nontarget lateralization	−0.049	0.191	0.095
N2ac	0.287*	0.072	0.084

Target Ta, target deviants elicited lateralized Ta effect; Nontarget Ta, nontarget deviants elicited lateralized Ta effect; Target Ta, target deviants elicited lateralized Ta effect; Target-minus-nontarget Ta, subtracting nontarget deviants from target deviants in lateralized Ta effect; Target lateralization, target deviants elicited lateralization effect in 200−350 ms; Nontarget lateralization, nontarget deviants elicited lateralization effect in 200−350 ms; N2ac, subtracting nontarget deviants elicited lateralization effect from target deviants elicited lateralization effect in 200−350 ms; *significance with correct (*p* value) < 0.05.

Table S4. The correlation between two types of ERP lateralization and behavior performance in TD children. (*r* value)

	RT	RT_{CV}	ERROR
50−150 ms			
Target Ta	−0.171	0.108	0.008
Nontarget Ta	−0.094	0.200	−0.055
Target-minus-nontarget Ta	−0.062	−0.076	0.052
200−350 ms			
Target lateralization	0.296*	−0.126	0.212
Nontarget lateralization	0.303*	−0.098	0.051
N2ac	0.019	−0.031	0.141

Target Ta, target deviants elicited lateralized Ta effect; Nontarget Ta, nontarget deviants elicited lateralized Ta effect; Target Ta, target deviants elicited lateralized Ta effect; Target-minus-nontarget Ta, subtracting nontarget deviants from target deviants in lateralized Ta effect; Target lateralization, target deviants elicited lateralization effect in 200−350 ms; Nontarget lateralization, nontarget deviants elicited lateralization effect in 200−350 ms; N2ac, subtracting nontarget deviants elicited lateralization effect from target deviants elicited lateralization effect in 200−350 ms; *significance with correct (*p* value) < 0.05.

Table S5. Correlation between the lateralization/N2ac effect and ADHD symptoms in children with ADHD (*r* value)

	$SNAP\text{-}IV_{inatt}$	$SNAP\text{-}IV_{hyper}$	$SNAP\text{-}IV_{full}$
Target lateralization	0.187	0.178	0.203
Nontarget lateralization	−0.136	−0.051	−0.098
N2ac	0.287*	0.216	0.276

$SNAP\text{-}IV_{inatt}$, inattention subscale of SNAP-IV; $SNAP\text{-}IV_{hyper}$, hyperactivity/impulsivity of SNAP-IV; $SNAP\text{-}IV_{full}$, full scale of SNAP-IV; Target lateralization, target deviants elicited lateralization effect in 200−350 ms; Nontarget lateralization, nontarget deviants elicited lateralization effect in 200−350 ms; N2ac, subtracting nontarget deviants elicited lateralization effect from target deviants elicited lateralization effect in 200−350 ms; *significance with correct (p value) < 0.05.

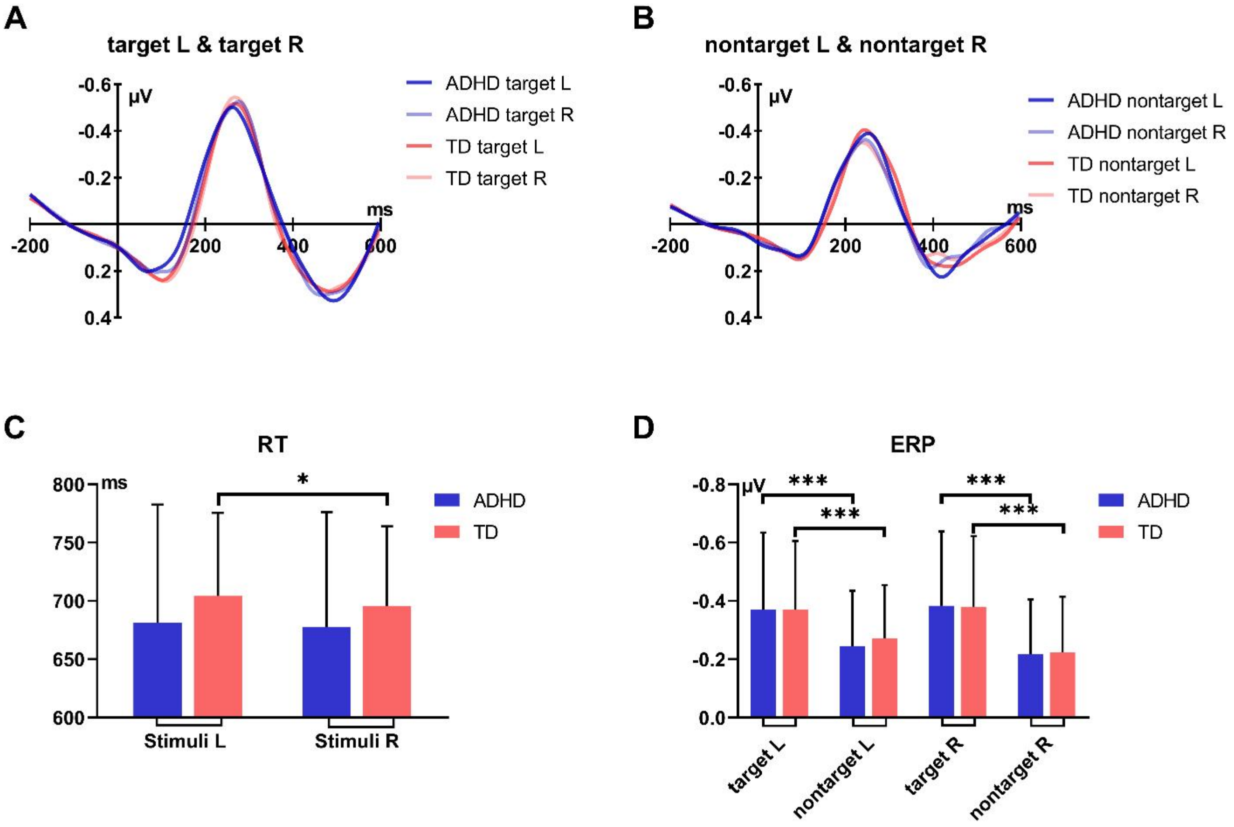

Figure S1. <u>Group differences between left and right stimuli.</u> (A) The grand-average ERPs to the target deviants of left side and right side. (B) The grand-average ERPs to the nontarget deviants of eft side and right side. (C) Statistical analysis results of RT. TD children showed slower RT when the sound played in the left than in the right, however, this effect was not observed in children with ADHD. (D) Statistical analysis results of ERP components (200–350 ms). No effect of Group × Stimulus Side × Condition was observed.
